# Supplementary material for: Empirical methods for controlling false positives and estimating confidence in ChIP-Seq peaks
Source: BMC Bioinformatics. 2008 Dec 5;9:523. doi: 10.1186/1471-2105-9-523 (PMC2628906; doi:10.1186/1471-2105-9-523)
Supplement: Additional File 1 — A variety of html documents from the USeq web site detailing the available applications, their best usage, output file type descriptions, command line menus, etc. [file 1471-2105-9-523-S1.zip › USeqUserGuides/JavaDoc.zip!/JavaDoc/gata/aligner/AlignerPreferences.html]

AlignerPreferences


|  |  |  |
| --- | --- | --- |
| **Overview** \| **Package** \| **Class** \| **Use** \| **Tree** \| **Deprecated** \| **Index** \| **Help** | |  |
| **PREV CLASS**   **NEXT CLASS** | **FRAMES**    **NO FRAMES**     **All Classes** |  |
| SUMMARY: NESTED \| FIELD \| CONSTR \| METHOD | DETAIL: FIELD \| CONSTR \| METHOD |  |


---


## gata.aligner Class AlignerPreferences

```
java.lang.Object
  gata.aligner.AlignerPreferences
```

**All Implemented Interfaces:**: java.io.Serializable

---

``` public class AlignerPreferences extends java.lang.Object implements java.io.Serializable ```

**Author:**
:   nix
    Container class to hold aligment preferences, this is saved after running GATAligner as a
    serialized object and used in subsequent GATAligner runs so the user doesn't have to reset
    the defaults every run.

**See Also:**: Serialized Form

---

**Constructor Summary**

`AlignerPreferences()`


| **Method Summary** | |
| --- | --- |
| `java.lang.String` | `getBaseName()` |
| `java.lang.String` | `getBl2SeqProg()` |
| `java.lang.String` | `getCompSeq()` |
| `java.lang.String` | `getCompStart()` |
| `java.lang.Integer` | `getCreate()` |
| `java.lang.Integer` | `getExtend()` |
| `java.lang.String` | `getExtract()` |
| `java.lang.String` | `getMask()` |
| `java.lang.Integer` | `getMatch()` |
| `java.lang.Integer` | `getMisMatch()` |
| `java.lang.String` | `getRefSeq()` |
| `java.lang.String` | `getRefStart()` |
| `java.lang.String` | `getScore()` |
| `java.lang.String` | `getStorageLoc()` |
| `java.lang.String` | `getWindow()` |
| `void` | `saveAlignerPreferences()` |
| `void` | `setBl2SeqProg(java.lang.String string)` |
| `void` | `setPreferences(java.lang.String refSeq, java.lang.String compSeq, java.lang.String bl2SeqProg, java.lang.String storageLoc, java.lang.String baseName, java.lang.Integer match, java.lang.Integer misMatch, java.lang.Integer create, java.lang.Integer extend, java.lang.String mask, java.lang.String extract, java.lang.String window, java.lang.String score, java.lang.String refStart, java.lang.String compStart)` |
| `java.lang.String` | `toString()` |

**Methods inherited from class java.lang.Object**

`equals, getClass, hashCode, notify, notifyAll, wait, wait, wait`

**Constructor Detail**

### AlignerPreferences

```
public AlignerPreferences()
```


**Method Detail**

### toString

```
public java.lang.String toString()
```

:   **Overrides:**: `toString` in class `java.lang.Object`

---


### setPreferences

```
public void setPreferences(java.lang.String refSeq,
                           java.lang.String compSeq,
                           java.lang.String bl2SeqProg,
                           java.lang.String storageLoc,
                           java.lang.String baseName,
                           java.lang.Integer match,
                           java.lang.Integer misMatch,
                           java.lang.Integer create,
                           java.lang.Integer extend,
                           java.lang.String mask,
                           java.lang.String extract,
                           java.lang.String window,
                           java.lang.String score,
                           java.lang.String refStart,
                           java.lang.String compStart)
```

---


### saveAlignerPreferences

```
public void saveAlignerPreferences()
```

---


### getBl2SeqProg

```
public java.lang.String getBl2SeqProg()
```

---


### getCompSeq

```
public java.lang.String getCompSeq()
```

---


### getCreate

```
public java.lang.Integer getCreate()
```

---


### getExtend

```
public java.lang.Integer getExtend()
```

---


### getMask

```
public java.lang.String getMask()
```

---


### getMatch

```
public java.lang.Integer getMatch()
```

---


### getMisMatch

```
public java.lang.Integer getMisMatch()
```

---


### getRefSeq

```
public java.lang.String getRefSeq()
```

---


### getStorageLoc

```
public java.lang.String getStorageLoc()
```

---


### getCompStart

```
public java.lang.String getCompStart()
```

---


### getRefStart

```
public java.lang.String getRefStart()
```

---


### getScore

```
public java.lang.String getScore()
```

---


### getWindow

```
public java.lang.String getWindow()
```

---


### getBaseName

```
public java.lang.String getBaseName()
```

---


### setBl2SeqProg

```
public void setBl2SeqProg(java.lang.String string)
```

---


### getExtract

```
public java.lang.String getExtract()
```


---


|  |  |  |
| --- | --- | --- |
| **Overview** \| **Package** \| **Class** \| **Use** \| **Tree** \| **Deprecated** \| **Index** \| **Help** | |  |
| **PREV CLASS**   **NEXT CLASS** | **FRAMES**    **NO FRAMES**     **All Classes** |  |
| SUMMARY: NESTED \| FIELD \| CONSTR \| METHOD | DETAIL: FIELD \| CONSTR \| METHOD |  |


---
